# Thermal Conductivity of Detonation Nanodiamond Hydrogels and Hydrosols by Direct Heat Flux Measurements

**DOI:** 10.3390/gels7040248

**Published:** 2021-12-03

**Authors:** Liliya O. Usoltseva, Dmitry S. Volkov, Evgeny A. Karpushkin, Mikhail V. Korobov, Mikhail A. Proskurnin

**Affiliations:** Chemistry Department, Lomonosov Moscow State University, 119991 Moscow, Russia; usoltsevalilya@gmail.com (L.O.U.); eukarr@gmail.com (E.A.K.); mkorobov49@gmail.com (M.V.K.); proskurnin@gmail.com (M.A.P.)

**Keywords:** nanodiamonds, hydrogels, hydrosols, heat-flow measurements

## Abstract

The methodology and results of thermal conductivity measurements by the heat-flow technique for the detonation nanodiamond suspension gels, sols, and powders of several brands in the range of nanoparticle concentrations of 2–100% *w*/*w* are discussed. The conditions of assessing the thermal conductivity of the fluids and gels (a FOX 50 heat-flow meter) with the reproducibility (relative standard deviation) of 1% are proposed. The maximum increase of 13% was recorded for the nanodiamond gels (140 mg mL^−1^ or 4% *v*/*v*) of the RDDM brand, at 0.687 ± 0.005 W m^−1^ K^−1^. The thermal conductivity of the nanodiamond powders is estimated as 0.26 ± 0.03 and 0.35 ± 0.04 W m^−1^ K^−1^ for the RUDDM and RDDM brands, respectively. The thermal conductivity for the aqueous pastes containing 26% *v*/*v* RUDDM is 0.85 ± 0.04 W m^−1^ K^−1^. The dignities, shortcomings, and limitations of this approach are discussed and compared with the determining of the thermal conductivity with photothermal-lens spectrometry.

## 1. Introduction

The technological and biomedical applications of the dispersions (by hydrosols and hydrogels) of hydrophilic nanodiamond (ND) nanoparticles have become widespread [1,2,3,4,5,6] due to their capability to form stable aqueous sols and gels with a specific size distribution [7]. Due to the developed particle surface with various functional groups [8,9], it is possible to control the physicochemical properties of aqueous nanodiamond materials. Diamond has many unique properties, including a high thermal conductivity (up to 2300 W m^−1^ K^−1^) [7,10]. Stable ND hydrosols are claimed to be capable of increasing heat transport efficiency (nanofluids) due to the nanoparticle contribution to the overall thermal conductivity [7,11,12]. Nanodiamond hydrogels may serve as highly versatile platforms for the controlled functionalization and delivery of a broad spectrum of therapeutic elements [13] or biocompatible and biofunctional multilayer nanofilms [14]. Undoubtedly, the thermal properties of carbon allotropes are unique [10], but how greatly the clusters and structures formed in solution change the thermal conductivity is still an essential and multifactorial question. However, the heat-conducting properties of two-phase systems containing nanodiamonds have not been systematically studied, though they are required for understanding the efficiency and value of the corresponding materials and the parameters that affect the thermal conductivity. Moreover, such two-phase systems require additional accuracy in data collection and interpretation, especially in dealing with the thermal conductivity of the liquid phases.

Non-stationary (dynamic) thermal methods are most common for thermal conductivity assessment [11] due to the rapid measurements, relatively low costs, simple and portable equipment, and the adequate accuracy of 5%. However, the thermal conductivity is assessed indirectly by measuring thermal diffusivity with the density and heat capacity externally found. An alternative for the dynamic thermal methods is optical, namely photoacoustic and photothermal methods, which provide the fast and non-contact determination of thermal conductivity with good precision [11,15].

Steady-state methods for thermal conductivity [16] are based on measuring the temperature difference at a steady heat flow through the sample. The calculation of thermal conductivity is based on determining the ratio between the transferred heat per unit of time (heat flux) and the temperature gradient across the area perpendicular to the direction of heat propagation at a constant rate. Thus, such a calculation is more pronounced and does not require external data. However, to achieve steady-state conditions, sufficient time is needed (about an hour), which is the main disadvantage; besides, the commercial equipment is mainly intended for solid samples. Nevertheless, steady-state methods show better precision and accuracy (2 and 3%, respectively) than the dynamic techniques, and recently, commercial developments have appeared for measuring liquids and pastes. These are the heat-flow meters [17], a variant of the parallel-plate technique, with cells for liquids and gels. However, there are almost no data on their use for sols and the precision and accuracy of these instruments.

In this work, direct heat-flow measurements were applied for thermal conductivity measurements for concentrated (2–13% *w*/*w*) nanodiamond aqueous solutions, covering the range in which the sol-gel transition usually occurs. As stated [18], stable gel structures emerge when the concentration reaches 5–10% *w*/*w*, when a sharp increase in viscosity is observed. Thus, the paper sums up the main methodological aspects for sol, gel, and paste measurements using the heat-flow technique, thus expanding the concentration range to one even broader.

## 2. Results

### 2.1. Nanodiamond Hydrosols

As the main objective of this work is thermal conductivity measurement in aqueous media, at the initial stage we established the conditions for the correct and reproducible measurements of the thermal conductivity of water. The heat flow meter (FOX 50) directly provides the value of fluid thermal conductivity; it is sufficient to pre-conduct a two-thickness calibration using the Pyrex standard from which the cell is made. The correctness of the direct measurements of the liquids is a weak point of the heat-flow method [17]; without a standard, the data are biased. However, supposing the experimental conditions—first of all, the temperature difference between two plates and the precise control of liquid thickness (Appendix A)—are set up correctly, using relative measurements with the water as a standard to unbias the measurements are fully justified, and the growth of thermal conductivity relative to the water can be considered reliable.

It was shown [19] that the sol-gel transition occurs when the ND concentration exceeds 5–6% *w*/*w* for sols with a negative electrokinetic potential. Using a rotational viscosimeter gives an excellent opportunity to measure the rheological properties over the wide range of shear rates: at high concentrations, the viscosity decreases linearly with the shear rate, proving the sol-gel transition. The authors demonstrated the appearance of yield stress and also indicated the formation of some networks in concentrated sols [19]. When the yield stress is overcome, constant viscosity is observed (Newtonian behavior for sols with concentrations below 5% *w*/*w*). The precise determination of the concentration threshold for the sol-gel transition was not a goal for this study as we were focused on investigating thermal properties in a wide range of concentrations, including ND aqueous sols and gels. For such systems, it is vital to know the level of dynamic viscosity, which is crucial for the heat-transfer application of fluids.

Meanwhile, a qualitative thixotropy effect was observed too, and all the sols were exposed to ultrasonic treatment (with an immersed probe technique) for 30 s before the experiments [20]. As shown previously [21], a two-step gelation process can explain the sol-gel transition reversibility under mechanical impact. Firstly, primary clusters are gathered into less compact aggregates joined into a more compact gel network. The sol-gel transition is explained by forming a gel network from chains of faceted ND particles due to the electrostatic interaction of the facets [22,23]. All these peculiarities may affect the thermal conductivity of the sols, but this was not studied previously.

Rheological measurements of the ND hydrosols were made for the plate gap during the measurement of 1.00 mm. For the RUDDM (100 mg mL^−1^) and the RDDM (200 mg mL^−1^) brands, according to the amplitude sweep test, the shear moduli of the dispersions were below the instrument detection limit (measured values *ca.* 0.1 Pa at 1 Hz). Therefore, from a rheological viewpoint, these samples are liquids with negligible contribution from the elastic behavior.

**Figure 1:**
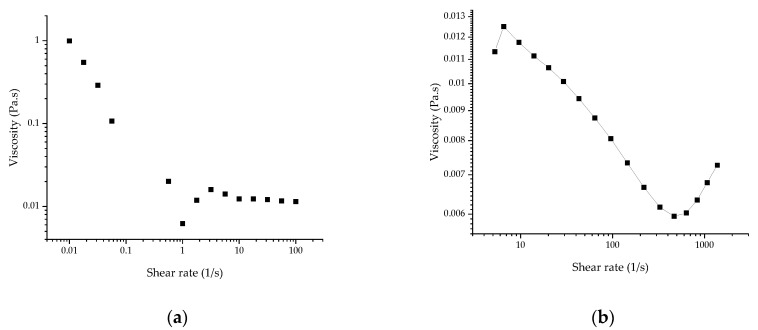
Viscosity curves of (**a**) RUDDM, 100 mg mL^−1^ and (**b**) RDDM, 200 mg mL^−1^ hydrosols of nanodiamonds.

**Figure 2:**
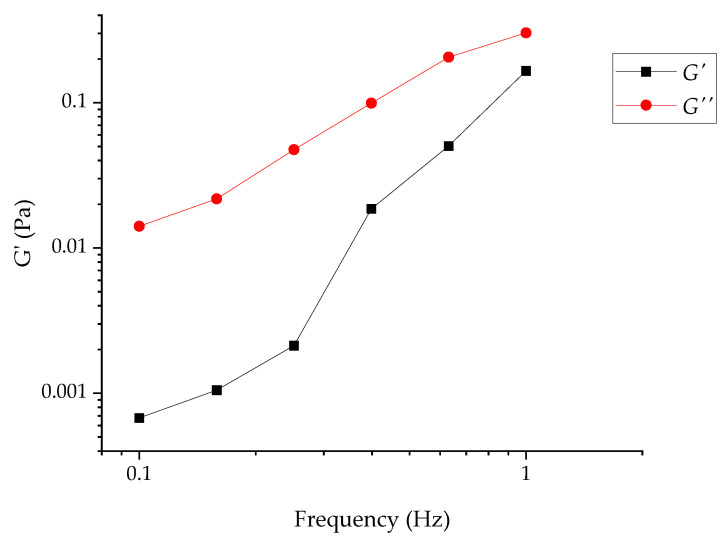
*G*′ and *G*″ moduli frequency tests for RDDM (200 mg mL^−1^).

For the RUDDM (100 mg mL^−1^), the measurement of the stationary viscosity revealed the pseudoplastic behavior of the dispersion (Figure 1). Because of the composition, it could be attributed to the disaggregation of the interacting particles of the dispersed phase under the action of shear stress. No sign of yield stress was observed, at least down to the lowest shear stress applied during the experiment (*ca.* 0.01 Pa). The measured values are approximate, due to the use of the parallel-plate arrangement (accurate determination of viscosity requires the use of a cone-plate cell, which was beyond the framework of this study). For the RDDM (200 mg mL^−1^), the attempt to assess the elastic behavior magnitude was unsuccessful. The reliable values of the moduli were obtained only at a reasonably high shear strain (3.00), which was definitely outside the linear rheology range. Moreover, the data at frequencies above 1 Hz were meaningless due to the resonance between the sample and the measuring system. The very approximate data which could be collected confirmed the predominantly viscous behavior of the sample as the *G*′ modulus was significantly lower than the *G*″ (Figure 2).

Tentative estimation of viscosity (Figure 1) showed that it was much lower for the RDDM than in the RUDDM samples. Instead of the lowest Newtonian viscosity plateau at the highest shear rates, it revealed a sign of dilatant behavior, which is less typical than the pseudoplastic behavior observed at a lower shear rate range but is not uncommon for concentrated fluid dispersions. Hence, the observation of the mixed pseudoplastic-dilatant behavior for that sample was tentatively in line with its higher concentrations.

Following the rheology tests, all the hydrosols were exposed to ultrasonic treatment (with an immersed probe technique) for 30 s before the experiments as we considered the sols as potential actively moving nanofluids. We did not see any severe effects on the thermal conductivity for the concentrated sols; the data immediately after cell loading and after an hour provided insignificantly different values.

**Figure 3:**
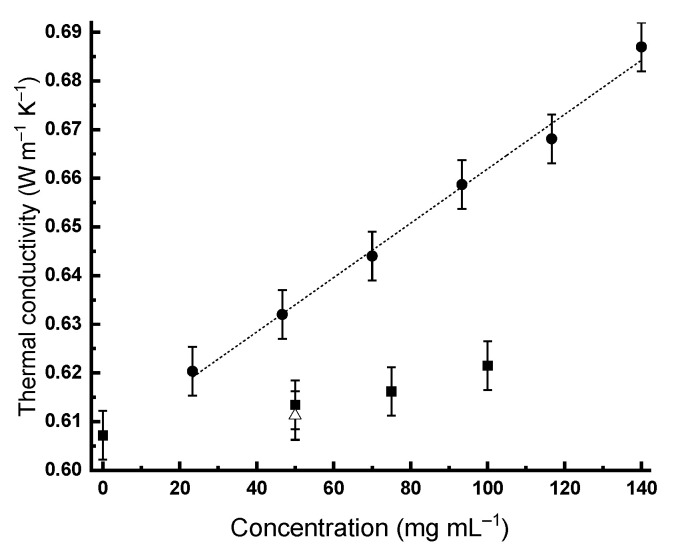
Thermal conductivity at 25 °C vs. the concentration of nanodiamond brands: RDDM, circles; RUDDM, squares; and SDND, triangle.

**Figure 4:**
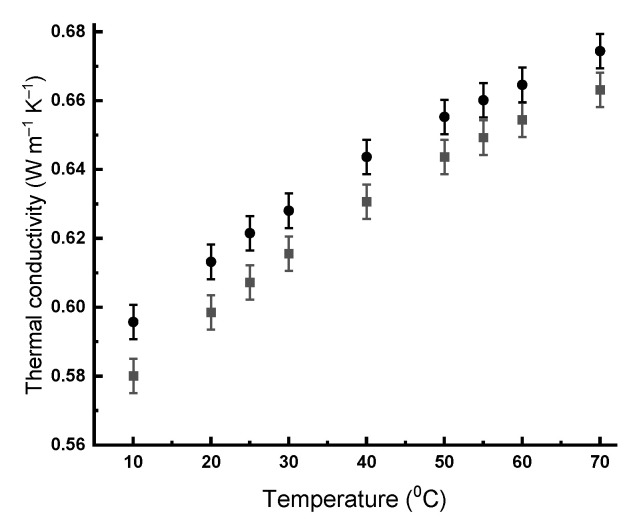
Temperature dependence of thermal conductivity of RUDDM hydrosols, 100 mg mL^−1^, circles, and water, squares.

If the experimental thermal conductivity of the colloidal solution significantly (taking into account the error of the method) differs from the experimental thermal conductivity of water, one can speak about a reliable increase in thermal conductivity. Thus, the concentration dependences of the thermal conductivity of the nanodiamond hydrosols were obtained (Figure 3). Figure 4 shows the temperature dependence of the thermal conductivity of the RUDDM solutions shifted up, as is typical for water. The same was observed for all the other aqueous sols.

The concentration of the commercial sample of SDND is 50 mg mL^−1^; for these solutions, thermal conductivity, taking into account the method error, slightly differs from the values characteristic to water (Figure 3). The same is observed for the RUDDM; a further increase in concentration leads to a slight increase in thermal conductivity (Table 1).

Interestingly, for similar concentrations, the thermal conductivity of the RDDM sols is higher than the others (Table 1). Moreover, this brand can form more concentrated stable gels due to the high solubility, which allows the observing of a linear dependence of the thermal conductivity on the concentration (in mg mL^−1^, which is the mass of particles divided by the volume of the solution and if divided by the (constant) density of particles gives the volume fraction) up to 140 mg mL^−1^ (4% *v*/*v*):

(1) $$k_{\mathrm{RUDDM}}=\left(56\pm 6\right)\cdot 10^{-5}c+\left(0.606\pm 0.006\right)$$

Further dilution below 20 mg mL^−1^ for the RDDM, or 50 mg mL^−1^ for the other brands, leads to values insignificantly different from water thermal conductivity. The results of the estimations of the different behavior of the thermal conductivity of the RDDM ND brand are in good concordance with the data of the photothermal measurements of the thermal conductivity of these brands, showing the maximum increase for this brand [15].

As a first approximation, to calculate the thermal conductivity of the two-phase systems, Maxwell’s theory is used, according to which [16,24] the increase in the thermal conductivity of sol (nanofluid) $k_{nf}$ over the base fluid $k_{bf}$ is determined by the volume fraction of nanoparticles $\varphi_{np}$ and the constant $x=k_{np}/k_{bf}$, where $k_{np}$ is the thermal conductivity of nanoparticles:

(2) $$k_{nf}/k_{bf}=1+\frac{3\varphi_{np}\left(x-1\right)}{\left(x+2-\varphi_{np}\left(x-1\right)\right)}$$

In this case, a rough estimation for the RDDM gives the value no higher than 30 W m^−1^ K^−1^. On the other hand, concentrated dispersions (50 mg mL^−1^ and above), for which the thermal conductivity is significantly different from the base fluid, as shown previously [25], have high viscosity values (from 1.5 times higher than that of water). It is noteworthy that the relative increase in dynamic viscosity compared to the base fluid can exceed the increase in thermal conductivity by no more than four times, in which case the addition of nanoparticles is practical [26].

The methodology of the measurements of sols with the heat-flow method was checked using silicon oxide sols (Appendix B) because they provide sols of a different nature with approximately the same expected thermal conductivity increase. For these substances, previous data exist [27,28,29]. The results show a good agreement of the data obtained for the silicon oxide of the different brands, good reproducibility of the data, and agreement with the previous studies (Appendix B). These results show that these sols can be used as reference materials for studies involving colloidal solutions and probably fulfill the lack of standards for thermal conductivity in liquids, especially two-phase liquids.

### 2.2. Nanodiamond Gels and Pastes

**Figure 5:**
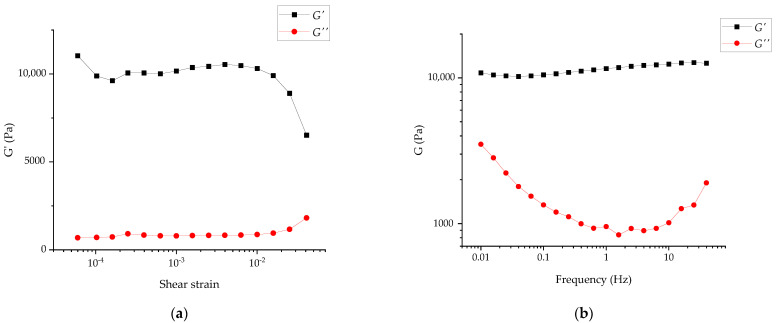
Amplitude (**a**) and frequency sweeps at the strain of 0.001 (**b**) for RUDDM, 200 mg mL^−1^.

Rheological measurements of the hydrogels were made in the forced oscillation mode only. The amplitude sweep for the RUDDM (200 mg mL^−1^) revealed the onset of nonlinear behavior at a strain of *ca.* 0.01. Therefore, the frequency sweep experiments were performed at the strain of 0.001, corresponding to a balance between a poor signal-to-noise ratio at low strain amplitudes and nonlinear behavior at high strain amplitudes, as in Figure 5a. In addition, the frequency sweep test revealed the predominance of the elastic response of the specimen over the entire probed range of frequencies 0.01 to *ca.* 50 Hz, as in Figure 5b.

Thus, the RUDDM sample could be classified as a physical gel that forms an elastic network of contacts between nanoparticles. An increase in the viscous contribution at low frequencies corresponds to the slow movement of particles (relaxation) in the network on a long timescale (fluidity). However, the constant value of the elastic modulus confirmed that elastically active contacts are not much disrupted during the relaxation. In general, the observed behavior is quite typical of concentrated suspensions of particles, the contacts between which are reversible but stable and strong enough. 

**Figure 6:**
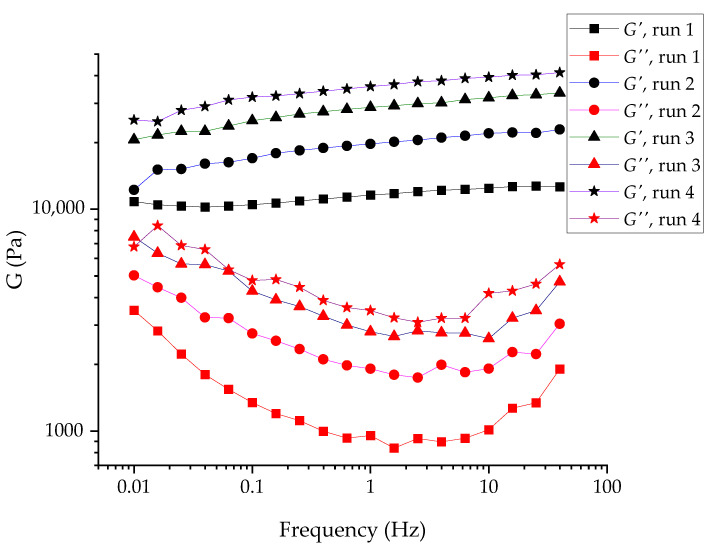
Successive frequency sweep measurements of RUDDM, 200 mg mL^−1^. Runs 1 to 4 correspond to 1, 2, 2.5, and 3 h aging of the gel upon the loading; each curve was recorded during the 25–30 min starting from high frequencies.

It should be noted that the structure of a physical gel is inevitably dispersed during the specimen loading to a measurement cell; therefore, it is always crucial to monitor the structure recovery. That was accomplished by successive frequency sweep measurements of the loaded specimen; see Figure 6. The figure shows that the structure recovery of the specimen was not yet complete even after 3 h of ‘resting’ (performing an oscillation test at low amplitudes), even though its rate was decreased significantly. The difference in the moduli measured during the first two runs was *ca.* 70%, whereas the comparison of runs 3 and 4, Figure 6, showed only a *ca.* 20% increase in the modulus.

**Figure 7:**
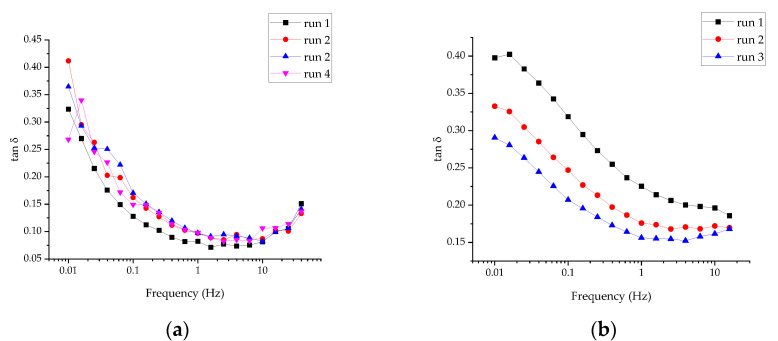
Tangent of the phase shift angle (the ratio between elastic *G*′ and viscous shear *G*″ moduli) for RUDDM, 200 mg mL^−1^ (**a**) and SDND, 100 mg mL^−1^ (**b**). Runs 1 to 4 correspond to 1, 2, 2.5, and 3 h aging of the gel upon the loading; each curve was recorded during the 25–30 min starting from high frequencies.

Whereas the moduli values reflected the overall “strength” of the gel (or the number of contacts per unit volume), their nature could be better reflected in the tangent of the phase shift angle (the ratio between *G*′ and *G*″); see Figure 7a. The change in tan δ values was negligible between runs 2–4; hence, the evolution of the sample upon the initial structure disruption could be described as an increase in the number of contacts between the particles without a significant change in the contact nature (e.g., strength or reversibility).

**Figure 8:**
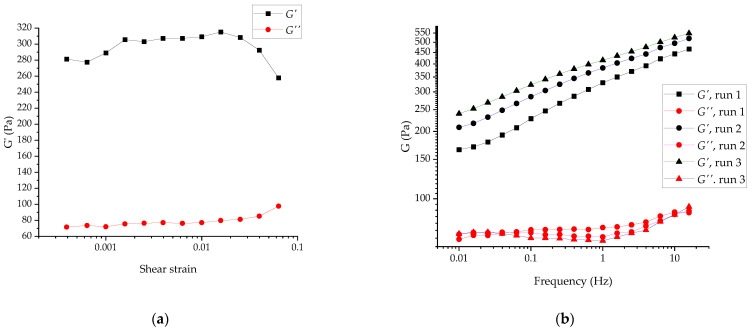
Amplitude (**a**) and frequency sweeps at the strain of 0.001 (**b**) for SDND, 100 mg mL^−1^. Runs 1 to 3 correspond to 1, 2, and 2.5 h aging of the gel upon the loading; each curve was recorded during the 25–30 min starting from high frequencies.

For the SDND (100 mg mL^−1^), the amplitude sweep test revealed the onset of the nonlinear rheological (structure breakup) behavior at a strain above 0.01. The initial increase in the moduli values was due to the recovery of the structure disrupted during the sample loading, which was not complete upon the initial rest (5 min); see Figure 8a. The frequency sweep tests were performed at a shear strain of 0.003, in the linear viscoelastic range; see Figure 8b. The difference in the moduli between runs 2 and 3 (corresponding to about 1.5 and 2.5 h upon the specimen loading, as in Figure 8b) was 8–10%. Hence, it could be roughly concluded that the SDND structuring was faster than that of the RUDDM. Other features special about the SDND overall frequency-sweep curves are: (i) the relative independence of the viscous shear modulus *G*″ on the frequency and prominent dependence of the elastic modulus *G*′ and (ii) the evolution of the tan δ curve during the structure buildup; see Figure 7b. Whereas recovery of the RUDDM structure was not accompanied by a significant change in the tan δ, meaning virtual independence of the contact nature, the prominent decrease in tan δ for the SDND showed the increased relative contribution of the elastic response to its rheological behavior. That could mean the strengthening of the contacts between the particles. However, the elastic network structure remained relatively weak even upon 3 h recovery at rest, as is evident from the elastic-modulus dependence on frequency.

**Figure 9:**
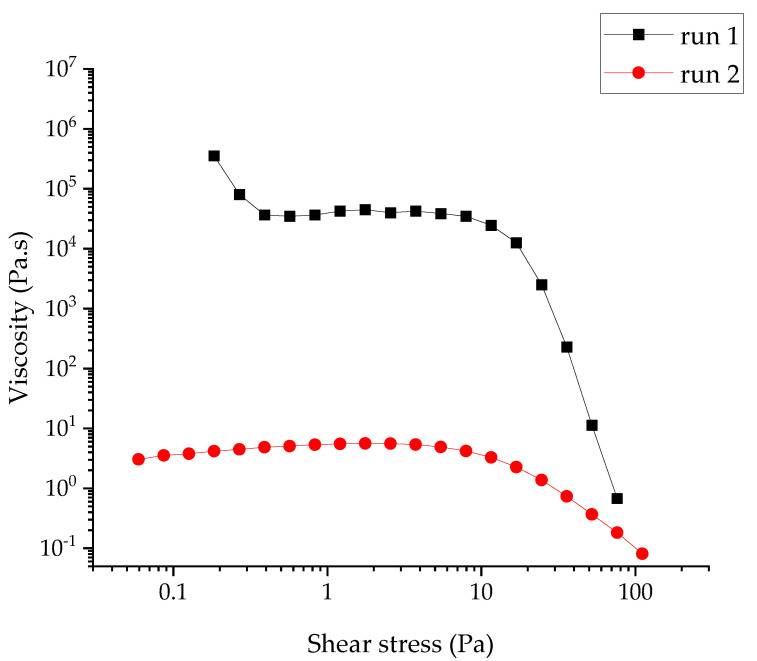
Viscosity curves for SDND, 100 mg mL^−1^. Curve 1 was recorded upon 3.5 h at ‘resting’, a small-amplitude oscillation test, and curve 2, immediately after curve 1.

The reversible structure rupture and buildup were confirmed in the viscosity curves for the SDND; see Figure 9. The shape of curve 1 marks the onset of apparent yield stress at a low shear stress of *ca.* 0.5 Pa due to the formation of the network of contacts, which is weak but spans the entire sample. The plateau of stress-independent (and relatively high) viscosity at 1–10 Pa is due to a slow flow induced by the applied stress, consisting of the movement of the particles via the reversible rupture of the contacts. In other words, the macroscopic network structure remained unchanged and gave the same resistance to the applied stress independently of its magnitude. When the applied stress exceeded 10 Pa, the network rupture became irreversible at the experiment timescale, and the viscosity decreased by four orders of magnitude over a narrow range of shear stress; see Figure 9. The network structure was not recovered immediately upon the flow at high shear stresses, as evidenced by the significantly lower viscosity values in curve 2 (Figure 9) and the disappearance of the yield stress onset at the lowest shear stress.

Thus, from the rheological viewpoint, the ND hydrogels resemble charged polystyrene latexes, in which the rheology depends on the concentration, particle size, and polydispersity, but have a weak relative overall structure [30]. Thus, various brands show different behavior despite the similarity in other properties.

Concerning the heat-flux measurements, due to some practical difficulties the reproducibility of measurements in a paste-specimen cell is lower than the reproducibility for solid materials and liquids. For the two-thickness test, the gel is placed twice into the cell, first with a thin and then with a wide spacer ring, and each time the system is completely disassembled. Therefore, it is necessary to ensure an even distribution of the sample, monitor the absence of bubbles, control the sample volume, remove excess protruding through release holes, and check the thickness of the layer. In this case, only a 10–15% accuracy was achieved for the approximate assessment of the paste thermal conductivity. In the case of gels, the cell loading was made as gently as possible to minimize the thixotropic effects described above; the measurement was started right after loading. In the case of even more concentrated pastes, the immediate measurement after loading was dictated by the relatively fast drying of the pastes, which affects the thermal conductivity coefficient to a great extent.

Checking the correctness of the measurement of gels in a paste specimen cell is quite tricky as certified materials of the thermal conductivity of pastes do not exist. As a reference sample, a commercial organosilicon thermal gel KPT-8 was used. According to GOST 19783-74 State Standard (Russia), its thermal conductivity at 20 °C is no less than 0.7 W m^−1^ K^−1^; according to the manufacturer’s data, the thermal conductivity is 0.7–0.8 W m^−1^ K^−1^. The experimental thermal conductivity of this sample at 25 °C was 0.89 ± 0.09 W m^−1^ K^−1^.

Compared to the sols, an increase was recorded for the nanodiamond gels; see Table 1. The maximum increase of 13% was reached for 140 mg mL^−1^ (4% *v*/*v*) of the RDDM brand. However, it is problematic to compare thermal conductivity data to the starting nanodiamond materials as they are powders with different size distributions, porosity, and purity. Therefore, only for estimation, the paste-specimen cell was used for the RUDDM and RDDM powder measurements (we estimated the nanodiamond relative density—the ratio of the apparent density of the powder to the solid bulk [15,25]—as 15–17%, so the porosity was 83–85%). At 25 °C, the thermal conductivity was 0.26 ± 0.03 and 0.35 ± 0.04 W m^−1^ K^−1^, respectively, which was consistent with the existing data [31]. However, it was impractical to measure a compressed sample as it was impossible to make a tablet of a nanodiamond powder with a diameter of 50 mm and a thickness of, e.g., 3–7 mm without a binder.

**Figure 10:**
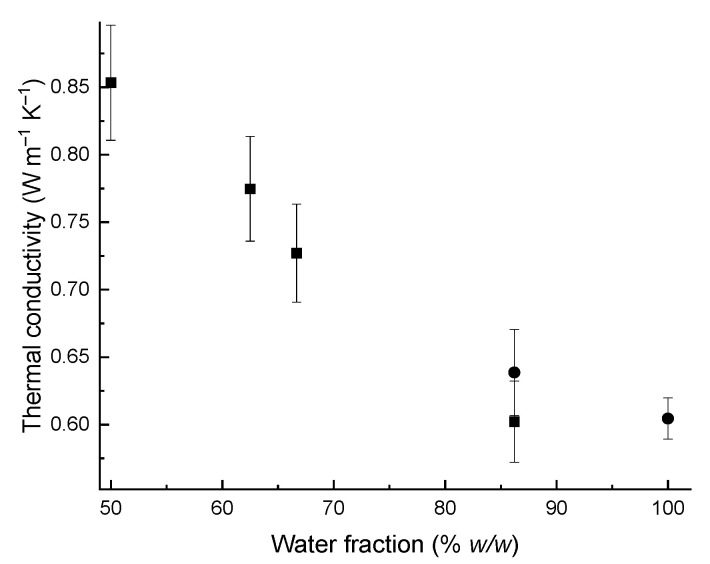
Experimental thermal conductivity values of aqueous RUDDM pastes and gels at 25 °C. Squares, measurements in the paste-specimen cell; circles, in the liquid cell.

Pastes were made to level the effect of the air gap on the heat-conducting properties of the powder by filling out the voids with water. For the paste measurements, the paste-specimen and fluid cells were used simultaneously for the most diluted samples (Figure 10).

For the paste obtained by mixing RUDDM powder with water in a mass ratio of 1:1 (which corresponds to the nanodiamond concentration of *ca.* 0.6 g mL^−1^ or 26% *v*/*v*), the thermal conductivity was low (0.85 ± 0.04 W m^−1^ K^−1^). According to Maxwell, assessing the thermal conductivity of nanodiamond gives an approximation for the RUDDM that is no more than 3 W m^−1^ K^−1^.

## 3. Discussion

Among the recent studies on the thermal conductivity of nanodiamond dispersions, inconsistent and fragmented data on its increase relative to the base fluid are observed [32,33,34,35,36,37,38,39,40]. As a result, there is no clear understanding of the reasons behind the thermal conductivity growth and whether it is worth expecting high values for nanodiamond dispersions [11]. The authors often refer to the high thermal conductivity of diamond (up to 2300 W m^−1^ K^−1^) [10], which should be much higher than the thermal conductivity of nanodiamond [31,41,42]. In principle, to transfer the thermal properties of bulk materials onto the nanostructures is not entirely correct as the contact resistance acquires primary importance, and the thermal conductivity mode may vary from diffuse to quasi-ballistic [43].

Furthermore, as nanodiamond is powdered, its thermal properties are associated with the surface contacts of the particles and the thermal conductivity of the gas gap [44]. As a result, the thermal conductivity of the powders is significantly lower (10–100 times) than that of the corresponding solid material; e.g., for a commercial sample of titanium oxide powder, thermal conductivity values of an order lower than for the bulk solid material [17] were obtained for almost similar measurement conditions. For relative densities below 80% and the small (<100 μm) size of the particles, the heat flux is regulated by the inter-particle contact resistance, which is often so large that metal powders can have the same heat-insulating properties as ceramic powders [45].

According to the existing data, an increase in the thermal conductivity of aqueous ND sols (up to 5% *v*/*v*) against water is 5–20% [32,33,35,46,47,48,49], but the data are quite contradictory, which prevents a comparative analysis. In this work, the data from two fundamentally different methods were compared to assess the thermophysical properties of the dispersions. The data on the heat-transfer parameters of the aqueous detonation nanodiamond dispersions of various brands by confocal photothermal microscopy and transient photothermal-lens modalities [15] showed that a 1 to 5% increase in thermal diffusivity and thermal conductivity relative to water was observed for ND concentrations of 1–4 mg mL^−1^. Unfortunately, the photothermal spectroscopy in the case of colloidal systems allows operation only in a narrow concentration range due to the interfering effect of light scattering. Thus, for nanodiamond sols, the upper limit is 5 mg mL^−1^ (0.5% *w*/*w* or 0.2% *v*/*v*).

According to heat-flux method measurements, the thermal conductivity of diluted ND sols is insignificantly different from the thermal conductivity of water. However, the calculated thermal conductivity values from the experimental data obtained using thermal-lens spectrometry showed an increase of up to 5% relative to the water [15]. Based on the results of the photothermal measurements, it can be assumed that nanodiamonds have a high thermal resistance at the cluster boundary [50]. Therefore, when the heating of the diluted sample is implemented thermally, heat transfer is carried out only through the water. In a photothermal experiment, the nanoparticles were heated due to their absorption of laser radiation [51]. After this, the particles stayed heated and released heat to the environment, leading to significant differences in the curves from the case of a homogeneous system [15,52]. It can also be assumed that in this case, it is more significant to contribute to the thermal conductivity by the mechanism proposed for nanosystems: diffusion of superheated or supercooled particles [53]. Due to the thermal resistance at the boundaries, the temperature of the particles may differ from the temperature of the surrounding liquid; they may be overheated. The contribution to heat transfer by such a mechanism is negligible for a liquid containing nanoparticles, but may increase in a thermal-lens experiment [15], which could be interpreted as the increase in the temperature of the whole environment [51].

Interfacial thermal resistance should affect heat transfer in a solution containing nanoparticles [50]. The typical resistance value, (0.3–1)∙10^−8^ m^2^ K W^−1^, depends on what the lyophilic or lyophobic surface is and, considering the value of the thermal conductivity of water, the contribution of thermal contact resistance should be taken into account for systems with particle sizes of *ca.* 2–6 nm [54]. Molecular-dynamics methods showed that ordered layers of water molecules around nanoparticles decreased contact resistance [55]. Therefore, the more efficiently the basic liquid “attracts” to particles and wets them, the less the resistance is. Accordingly, if the surface of the nanoparticles is covered with hydrophilic groups, the contact resistance is less than for a hydrophobic system [56]. In addition, it was shown that the local thermal conductivity of the aqueous shell adjacent to the nanoparticle is approximately 50% higher than in volumetric water [57]. The calculations were made for metal particles [56,57] and systems containing graphene (4∙10^−8^ m^2^ K W^−1^) [55,58] and carbon nanotubes [59].

The RDDM material has a higher thermal conductivity than other brands. First, the estimation of the thermal conductivity of nanodiamond phase for this brand by the linear dependence of the thermal conductivity of the two-phase system from the volume fraction of particles according to Maxwell [60] is not more than 30 W m^−1^ K^−1^, which is an order above the value for the RUDDM. Moreover, the RDDM differs in several properties from other nanodiamond samples, although according to X-ray diffractometry of the RDDM and RUDDM, the reflexes correspond to the crystallographic planes of the diamond cubic crystal lattice [61]. No extraneous phases were detected in significant amounts (more than 5%) (Supplementary Materials). The IR spectra of the brands are very close and contain absorption bands of groups: –OH (adsorbed water, surface OH groups, and hydrogen bonds), –C=O, and –COH (carboxyl and other groups) [61]. Aqueous dispersions of these brands have high values of electrokinetic potential, and the maximum is for RDDM (−66 mV), which is also characterized by the highest solubility [61].

According to the manufacturer, RUDDM is obtained by an explosion of a mixture of TNT and hexogen, while RDDM is of a mixture of graphite and hexogen, which can explain the unique optical properties of the latter [62]. Thus, as previously discussed [62], the aqueous dispersions of RDDM are characterized by maximum light absorption, probably resulting from more extensive sp^2^ layers and significant light scattering due to the presence of large particles noticeable on photothermal microscopic measurements [15]. The metal content is minimal for the RDDM brand (total, 1 mg g^−1^) [12]. The surface area of the mesopores according to BET for the RUDDM brand is fivefold higher (339 m^2^ g^−1^) than for RDDM (56.7 m^2^ g^−1^) [61], but the area of micropores is greater for RDDM (6.9 and 2.0 m^2^ g^−1^, respectively), and they are responsible for the hydrophilicity of the surface of nanodiamond, which is provided by the surface groups and adsorbed water [61]. The latter factor may cause lower contact thermal resistance for RDDM and increase thermal conductivity.

## 4. Conclusions

Thus, according to the heat flux method, this study shows that the thermal conductivity of the diluted sols of nanodiamonds differs from the thermal conductivity of water slightly, and significant (from 4%) growth is observed for concentrated (from 50 mg mL^−1^, 1.7% *v*/*v*) nanodiamond hydrogels. Pastes obtained by mixing water and nanodiamond powder also have relatively low thermal conductivity (0.85 ± 0.04 W m^−1^ K^−1^ for a paste containing 26% *v*/*v* RUDDM). Therefore, a high increase in thermal conductivity expected from diamond thermal conductivity is not reached. Previous thermal-diffusivity measurements by photothermal spectroscopy supported the data of sols and gels by direct heat-flow measurements [15]. The combined use of time-resolved thermal-lens spectrometry, photothermal microscopy, and the heat flow method showed that aqueous dispersions of nanodiamonds manifest themselves as photothermal heat-accumulating materials. This property of nanodiamond dispersions can be used in biomedical applications and needs to be further explored.

## 5. Materials and Methods

### 5.1. FOX 50 Heat Flow Meter

The FOX 50 Heat Flow Meter (TA Instruments, New Castle, DE, USA) was used to measure the thermal conductivity by the heat flux method defined by international standards ASTM C518, ISO 8301, and DIN EN 12667. In this compact analyzer, thin-film heat flux transducers made of dozens of small thermocouples are bonded to the surfaces of both plates, providing high sensitivity and signal integration. Type E thermocouples are bonded in the center of each transducer to give accurate readings of both plate temperatures. FOX 50 is equipped with a digital sample-thickness readout sensor and an integrated contact-resistance correction. When a sample is placed between the two plates under the action of compressed nitrogen, the lower plate moves up, and the guard caps are closed, providing a dense contact of the sample with the surfaces and good thermostatic conditions. To control the achievement of thermal equilibrium, the averaged data (columns of temperatures and sensor signals for the upper and lower plates) of one block (256 acquisition cycles) are compared to the respective average values of the previous block. If this comparison satisfies all the equilibrium criteria, the sample is declared to be thermally equilibrated, and the test is ended.

To measure the thermal conductivity of liquids, a specially made cell should be used (Supplementary Materials, Figure S1). The cell comprises two flat Pyrex glasses framed into the polyethylene terephthalate (PET) spacer ring. During compression, two O-rings used to seal the cell can shrink with varying degrees, thus resulting in different cell thicknesses. This parameter should be controlled to achieve reproducible results (Appendix A3).

A specially made cell for pastes (Supplementary Materials, Figure S2) comes with the subsequent model DTC 300 (TA Instruments); however, the cell can be adjusted to the FOX 50 instrument after some system modifications. It consists of two metal plates separated by a set of insulating rings. The inner ring is a spacer to hold the metal plates at a fixed distance. The outer ring is primarily a container for the sample material; each cuvette is equipped with two sets of rings, including thin and thick spacers for two-thickness tests.

### 5.2. Other Equipment

A commercially available off-the-shelf ultrasound probe with a timer is the MEF93.T (LLC MELFIZ-ul’trazvuk, Moscow, Russia). The ultrasound probe has an operating frequency of 22.00 ± 1.65 kHz, which works in a continuous mode of exposure to ultrasonic energy. In this work, an ultrasonic tip (surface area, 6.61 ± 0.02 cm^2^) was used, which provided an intensity range up to 250 W cm^−2^ in an electrical-power mode of 0.6 kW. The ultrasound tips were made of titanium alloys, grade TM3 (ISO 28401:2010). A GRAD 28–35 ultrasound bath from Grad-Technology (Russia) was used for preparing nanodiamond pastes. A SNOL 20/300 heating oven (Snol-Term Ltd., Tver, Russia) was used to evaporate sols. Other methods of the characterization of nanodiamond materials are summed up in the Supplementary Materials.

### 5.3. Samples

We used commercially available NDs, summed up in Table S1. For analysis, the nanodiamond powders were used as they were, or the nanodiamonds were gently ground in a jasper mortar.

### 5.4. Reagents and Solvents

Ethanol cp, 95%, Reakhim (Moscow, Russia); ultrapure water Milli-Q^®^ Type (Merck, Germany); N-methyl-2-pyrrolidone (NMP) 99.5%, Sigma Aldrich (St. Louis, MO, USA); methanol HPLC 99.8%, J.T.Baker (Philipsburg, NJ, USA); hexane for spectroscopy 99.5%, Component-reaktiv (Moscow, Russia); ethylene glycol 99.8%, Sigma Aldrich, (St. Louis, MO, USA); and heat-conducting paste KPT-8, 400 g (Connector, St. Petersburg, Russia) were used throughout.

### 5.5. Sample Preparation Procedures

#### 5.5.1. Preparation of Sols and Gels

RUDDM powder (5.000 ± 0.001 g) was placed into a 50 mL self-standing tube. Then, approximately 40 mL of deionized water was added. The solution was exposed to ultrasonic treatment for 1 min with the help of a probe. Then, the tube was filled to the mark with deionized water and subjected to another 30 s ultrasound treatment. RDDM powder (3.000 ± 0.001 g) was placed into a 50 mL self-standing tube. Then, approximately 20 mL of deionized water was added. The solution was exposed to ultrasonic treatment for 1 min with the help of a probe.

The concentrations of the stock solution were found by gravimetry. Then, a series of working solutions with the concentrations of 50−100 mg mL^−1^ for the RUDDM and 23−140 mg mL^−1^ for the RDDM were prepared.

Hydrosols for rheological measurements were also prepared by a high-powered continuous ultrasound probe, as described elsewhere [20]. Due to the small volume of the nanodiamond dispersion sample (up to 15 mL, 100–200 mg mL^−1^), a water-cooling circuit during sonication was used in this work for the first time.

#### 5.5.2. Preparation of RUDDM Pastes

A weighed portion of RUDDM powder was mixed with a known mass of deionized water with a spatula. Then, the glass beaker was transferred to an ultrasonic bath. An hour was needed to obtain a visually homogeneous paste with a fraction of water of 50.0−86.2% *w*/*w*. The most diluted sample was also prepared as described above with the help of a probe and measured in the liquid cell.

### 5.6. Determination of Thermal Conductivity

The principle of operation of the heat-flow meter is based on the one-dimensional equation for the Fourier–Biot law:

(3) $$q=-k\left(\frac{dT}{dx}\right),$$

where *q* (W m^−2^) is the heat flux through the sample, *k* (W m^−1^ K^−1^) is the sample thermal conductivity coefficient, and dT/dx is a temperature gradient on the isothermal flat surface of the sample.

The sample is placed between flat isothermal plates, hot and cold. As a result, a uniform one-dimensional thermal field is achieved throughout the volume of the sample, and the temperature gradient is equal to the temperature difference between the hot and cold surfaces of the sample, divided by its thickness, ΔT/Δx. The thermal resistance of the flat sample $R_{sample}$ (m^2^ K W^−1^) is its thickness divided by thermal conductivity:

(4) $$R_{sample}=\Delta x/k$$

For a sample with high thermal conductivity, the temperatures of its surfaces are not equal to the temperatures of the instrument plates due to contact resistance:

$$T_{cold\;plate}<T_{cold\;surface}<T_{hot\;surface\;}<T_{hot\;plate\;},\;\delta T+\Delta T_{sample}+\delta T=\Delta T_{plates}$$

The temperature jump is proportional to the heat flux and thermal contact resistance (δT=qR).

The electrical signal *Q* (μV) is proportional to the heat flux, q, which is equal to the temperature difference, $\Delta T_{plates}$, divided by the sum of the thermal resistances of the sample, Equation (4), and two contact resistances, 2R:

(5) $$Q=\frac{q}{S_{cal}}=\frac{\Delta T}{\left(\Delta x/k+2R\right)S_{cal}}$$

Tests using two same-material samples of different thicknesses ($\Delta x_{1}>\Delta x_{2}$) provide correct thermal conductivity coefficient values excluding the thermal contact resistance. Before tests, the instrument must be calibrated and the calibration coefficients for the upper and lower plate determined ($S_{cal.up}$ and $S_{cal.low}$). Two specimens of the same material and different thicknesses are used. Then, the system of two equations for heat-flow signals is resolved automatically by software to obtain two unknown values, $S_{cal}$ and 2R. In the case of the thermal insulators (large values Δx/k), the contact resistance can be neglected, but if *k* > 0.3 W m^−1^ K^−1^, it is necessary to use Equation (5). In the case of a liquid cell, Pyrex/instrument thermal contact resistances were used that are determined with a two-thickness calibration, $2R_{contact}$ (Appendix A and Section 5.7.1 below).

FOX 50 can measure only the total thermal resistance (liquid, two Pyrex glasses, and sample/instrument thermal contact resistances):

(6) $$R_{total}=R_{liq}+2R_{Pyrex}+2R_{contact}=\frac{\Delta T}{q}=\frac{\Delta T}{S_{cal}Q}$$

Thus, to calculate the liquid thermal conductivity, the software utilizes the equation:

(7) $$k=\frac{\Delta x_{liq}}{R_{total}-2R_{Pyrex}-2R_{contact}}=\frac{\Delta x_{total}-2\Delta x_{Pyrex}}{\Delta T/S_{cal}Q-2\Delta x_{Pyrex}/k_{Pyrex}-2R_{contact}}$$

### 5.7. Heat Flux Measurements

#### 5.7.1. Two-Spacer Calibration with Pyrex

For calibration, Pyrex was used, with thicknesses of 5 and 12.9 mm. The main parameters of the measurements are presented in Table 2. In addition, it was necessary to introduce thermal conductivity values for Pyrex at different temperatures; the table is provided with standards. Both the calibrations and the measurements in the liquid cell were carried out at 20, 30, 35, 40, 50, 60, 65, and 70 °C.

Calibration of the cell for paste specimens was carried out similarly, except that the Pyrex standards were placed not just between the instrument plates but also into the paste-specimen cell (Supplementary Materials, Figure S2).

#### 5.7.2. Measurements in a Liquid Cell

A liquid specimen cell was filled from the bottom with a syringe to let the air out of the cell. Next, the software calculated the thickness of the liquid layer by subtracting doubled glass thickness (3.3 mm) from the total measured value. At first, the water was tested, and then, sols with increasing concentration were examined successively without cell disassembling.

#### 5.7.3. Measurements in the Paste-Specimen Cell

In contrast to operating a liquid cell, measurements in the paste-specimen cell were carried out at two sample thicknesses, as in Equation (5). The sample was first placed in a cell with a thick (10.1 mm) spacer ring with a spoon or a spatula, filling the volume inside the inner ring until the sample rose slightly above ring level. Next, it was covered with an upper plate (Supplementary Materials, Figure S2); at the same time, excess paste came out through release holes and was removed with a napkin. After the measurement, the cell was emptied, a thin (4.9 mm) inner ring was inserted, filled with the same sample, and the test was performed again. The other parameters were similar (Table 2).

### 5.8. Rheological Measurements

Rheological measurements were performed using a parallel-plate measuring cell (diameter 35 mm) of the RheoStress 600 rheometer (Haake, Hadamar-Steinbach, Germany). The measurements were carried out at a constant temperature of 25.00 ± 0.05 °C, maintained using a Peltier element. The specimens were allowed to equilibrate for 10 min upon loading to the rheometer cell, if not stated otherwise.

Steady flow viscometry measurements were performed by stepwise variation of shear stress or shear rate, the measuring duration at each step being 60 s. That was enough for the stationary flow to be developed.

Forced oscillation measurements were performed either at a constant frequency of 1 Hz and a varied shear strain of 10^−5^–10 or a constant shear strain of 0.001–0.003 (determined from the amplitude sweep test) and a varied frequency of 0.01–100 Hz.

To prevent the evaporation of the sample and its contact with the atmosphere, the specimens were coated with a thin layer of low-viscosity mineral oil prior to the test. The oil viscosity was much lower than that of the dispersions; therefore, the oil layer contribution to the measured sensor displacement was negligible.

## Figures and Tables

**Table 1:** Experimental values of thermal conductivity (±0.005) of nanodiamond hydrosols at 25 °C, in brackets is an increase relative to water (*k* = 0.607 W m^−1^ K^−1^).

RDDM	*c*, mg mL^−1^	23	47	70	93	117	140
	*c*,% *v*/*v*	0.7	1.4	2.0	2.7	3.4	4.1
	*k*, W m^−1^ K^−1^	0.620 (2%)	0.632 (4%)	0.644 (6%)	0.659 (8%)	0.668 (10%)	0.687 (13%)
RUDDM	*c*, mg mL^−1^		50	75	100		
	*c*,% *v*/*v*		1.7	2.4	3.5		
	*k*, W m^−1^ K^−1^		0.613	0.616	0.621 (2%)		
SDND	*c*, mg mL^−1^		50				
	*c*,% *v*/*v*		1.4				
	*k*, W m^−1^ K^−1^		0.611				

**Table 2:** Measurement parameters of heat flow for cells.

Measurement	T.E.	S.E.	P.E.%	min N.B.	C.B.	Δ*T*
Two-thickness calibration	0.5 °C	200 µV	2	16	6	20 °C
Liquid cell test	0.5 °C	200 µV	2	15	5	20 °C

Parameters: the deviation of the average temperature of each plate (T.E.); the difference between sensor signals of two successive blocks (S.E.); the relative change in sensor readings of two successive blocks (P.E.); the number of blocks that must pass the previous equilibrium criteria (min N.B.); and the number of blocks for calculating results (C.B.).

**Figure A1:**
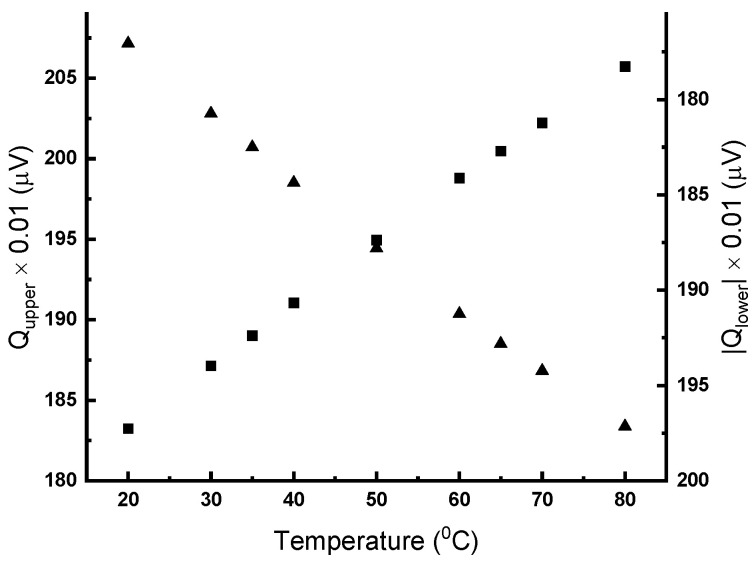
Signals (divided by 100) of heat flux sensors at different temperatures for a Pyrex standard at Δ*T* of 20 °C (squares for the upper and triangles for lower plates (the modulus for the lower plate is used).

**Figure A2:**
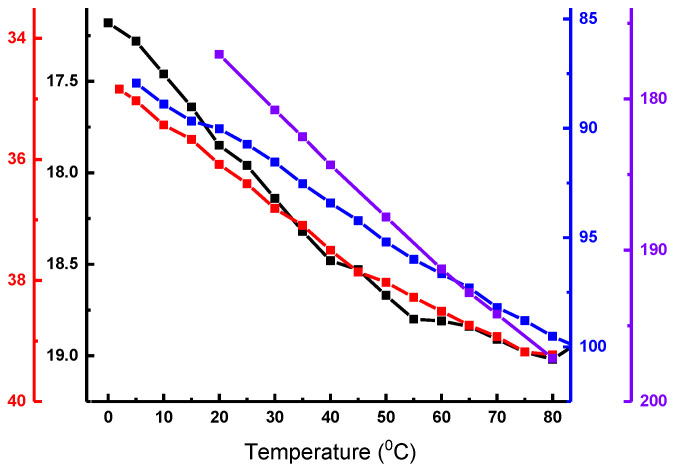
Signals (μV, modulus values divided by 100) of the lower plate of the heat flux sensor when measuring the Pyrex standard at different Δ*T* (2 °C, black; 4 °C, red; 10 °C, blue; and 20 °C, purple).

**Figure A3:**
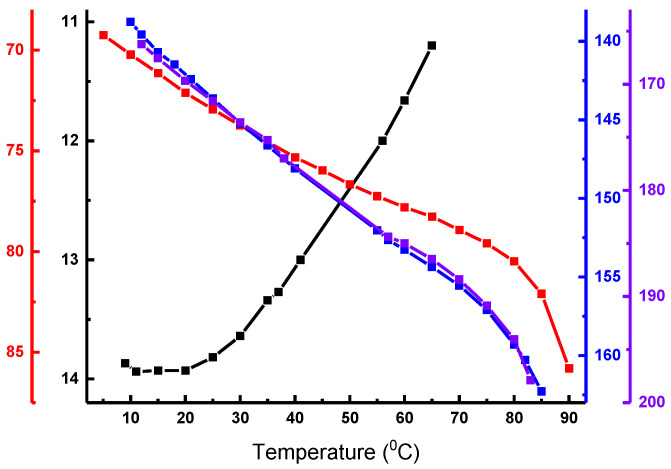
Signals (μV, modulus values divided by 100) of the lower plate of the heat flux sensor when measuring water at different Δ*T* (2 °C, black; 10 °C, red; 20 °C; and blue, 24 °C, purple).

**Figure A4:**
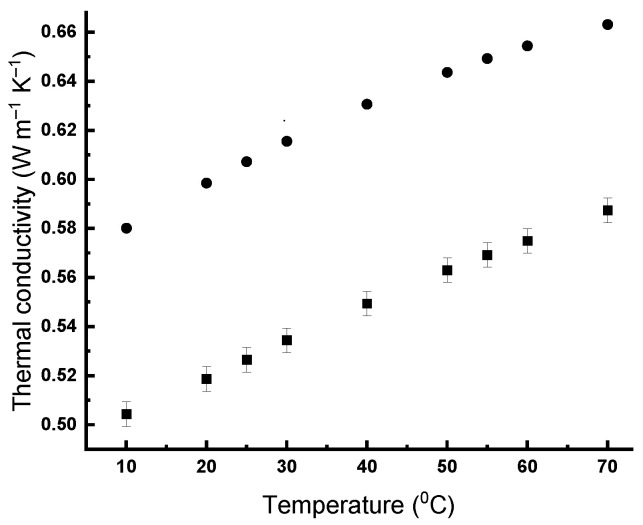
Thermal conductivity of water at different temperatures. Circles are plotted data taken from [63], squares are biased experimental data of 12 replicate measurements (RSD, 0.01).

**Figure A5:**
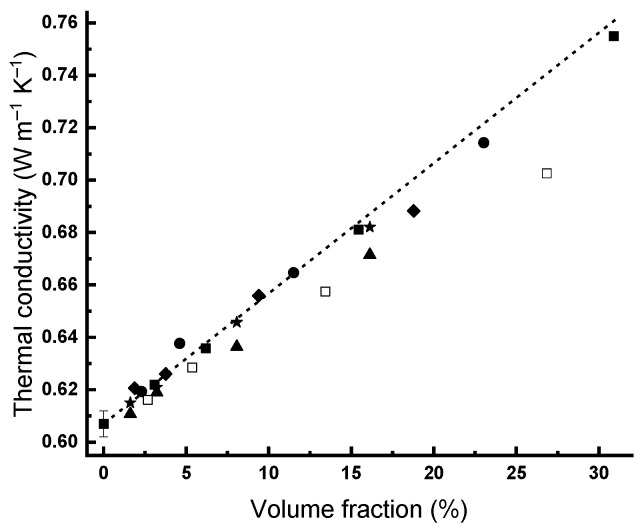
Experimental dependences of thermal conductivity of aqueous sols at 25 °C on the volume fraction of silicon oxide: AM, stars; SM-30, triangles; TMA, rhombs; HS-40, circles; CL-X, hollow squares; and TM-50, squares. The dashed line is theoretical values by the Maxwell equation ($k_{NP}\;$= 1.22 W m^−1^ K^−1^).

**Figure A6:**
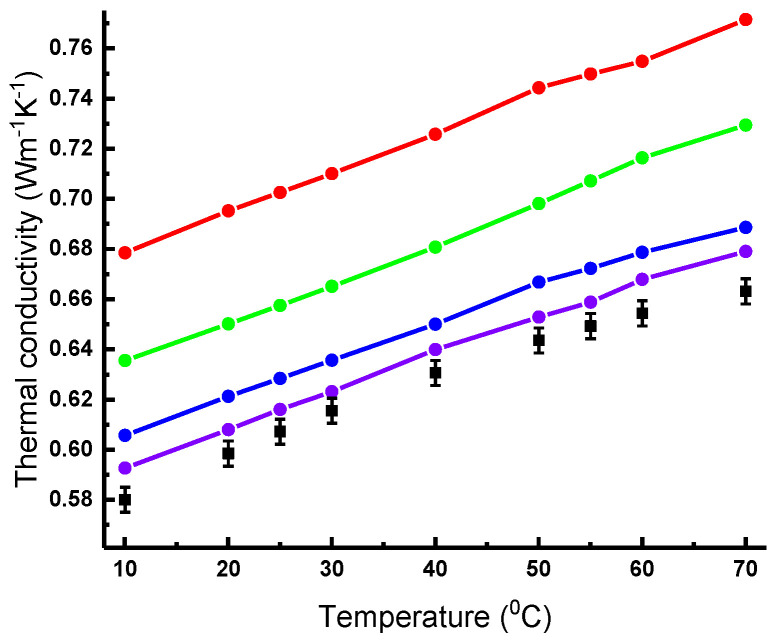
Experimental values of thermal conductivity of water (squares) and hydrosols of CL-X silicon oxide (circles): red, original solution; green, blue, and purple; solutions diluted 2, 5, and 10 times, respectively. The errors of measurements are the same and are shown for water only.

## Data Availability

Not applicable.

## Supplementary Materials

The following are available online at https://www.mdpi.com/article/10.3390/gels7040248/s1, Figure S1: cell for fluids of the FOX 50 analyzer, Figure S2: cell for paste specimens (top view) in a disassembled form (left) and a side view with a thin spacer (right), Figure S3: linear dependence of the experimental and reference thermal conductivity values for NMP, ethanol, methanol, ethylene glycol, and water, Figure S4: DLS curves for SDND and RUDDM samples (red and blue lines). The concentrations of nanodiamonds for all samples were 1.0 mg mL^−1^, Figure S5: ATR–FTIR spectra of different trademarks of ND. 1, RUDDM, fraction 0–0.15 μm; 2, SDND; 3, RDDM. Dashed drop lines correspond to bands of carbonyl and hydroxyl groups, Table S1: nanodiamond brands used in the study and estimated mean particle sizes from the Gibbs–Kelvin equation (*p* = 0.95, *n* = 3), Table S2: element contents in nanodiamond samples, determined directly in dispersions (all concentrations are in ppm, results uncertainty is ±15%), Table S3: functional groups on the surface of the studied nanodiamonds, Table S4: increase in the thermal conductivity of silicon oxide sols relative to water, Table S5: colloidal silicon oxide LUDOX brands.

## Appendix A. Calibration of the Heat Flux Cell for Liquids

### Appendix A1. Plate Temperature Difference

The temperature difference of the heat-flow meter’s upper and lower plates (Δ*T*) deserves special attention. Without explanation, the manufacturer indicates that the typical values are 10–20 °C, specifying that the temperature of the upper plate should be more than 20 °C higher than the lower for measurements in the liquid cell. The heat-flow meter measures the electrical signal on both plates, calculates two thermal conductivity values, and averages them. As the Pyrex standard and water thermal conductivities increase with temperature, the heat flux (the signal *Q*, Equation (5)) vs. temperature dependence should be almost linear. For the lower plate, *Q* is always below zero. As shown in Figure A1, when Δ*T* = 20 °C the signal modulus values are close for both plates and increase with temperature. However, if the measurements are carried out at lesser values of Δ*T*, the temperature dependence of the lower plate signal deviates from linearity (Figure A2).

The most characteristic changes occur if the low-temperature difference values are used when using a liquid cell. Figure A3 shows the heat flux sensor signals of the lower plate when measuring water in a liquid cell at different values of Δ*T*.

Note that the abnormalities were observed only for the lower plate signals. When the temperature difference is 2 °C, an uncharacteristic dependence is observed (the signal modulus decreases with temperature), leading to absurd conductivity data when averaging the results for the upper and lower plates. As a result, the liquid measurements were carried out with Δ*T* = 20 °C as the manufacturer states and in the temperature range of 10–70 °C, where the expected dependence was guaranteed.

### Appendix A2. Solvent

It is almost impossible to monitor the temperature dependence of heat fluxes, especially for the signals of the lower plate sensor for some organic solvents, as many solvents are aggressive for a liquid cell. For example, hexane, toluene, acetonitrile, NMP, and methanol interact with the sealing rings, violating the leak tightness. In addition to water (Figure A4), temperature dependences were constructed only for ethylene glycol; the heat flux modulus increased with temperature for both plates, as it should have. Other organic liquids were measured only at one point; for all cases, the experimental values were underestimated (Table A1) relative to the reference values with a constant coefficient (Supplementary Materials, Figure S3), probably due to a system design shortcoming that leads to some heat losses.

**Table A1:** Comparison of experimental and reference values of thermal conductivity of various liquids at 25 °C.

Solvent	*k*_exp_ ± 0.005	*k*_ref_ [63]
Water	0.526	0.607
Methanol	0.194	0.202
Ethanol	0.158	0.167
Ethylene glycol	0.233	0.254
N-methyl-2-pyrrolidone	0.157	0.167

### Appendix A3. Sample Thickness

The correct and reproducible value of thermal conductivity, according to Equation (7), is directly related to precise sample thickness determination. The main obstacle here is the variable shrinking of O-rings made from fluoroelastomer (Viton) under compression. However, if some precautions are fulfilled, this problem can be solved; namely, at the beginning of the test run, the sample thickness read should give the values around 5.04 ± 0.06 mm; usually, it was achieved when the nitrogen pressure was 5.0–5.5 atm, when the tight-fitting of glasses was reached. This parameter should be strictly the same for the water (standard) and the sols. For concentrated, viscous gels, this was accomplished by increasing the pressure to 6 atm. Otherwise, the growth of thermal conductivity relative to water could be overestimated by the higher thickness rather than the lower total thermal resistance. Furthermore, it is essential to use the same cell assembly in one experimental batch, starting with water and afterwards measuring sols with increasing concentrations without the cell disassembling. A very low (0.005 W m^−1^ K^−1^) method error was accomplished in such a case.

## Appendix B. Silicon Oxide Sols

Commercial sols of silicon oxide were measured by the FOX 50 analyzer to check the correctness of the proposed methodology for sols. Moreover, the thermal conductivity increase was significant enough for these systems to plot the volume dependences for different types (Figure A5).

As in the case of the nanodiamond sols, the existing data on the thermal properties of the silicon oxide sols are ambiguous (Supplementary Materials, Table S4). On average, for 3–5% *v*/*v* sols, a 3–5% relative increase in thermal conductivity was observed relative to water [27,64,65], which is well consistent with the theoretical calculations according to Maxwell, Equation (2) [27].

There is almost no explanation of some of the reported anomalous (above 20%) growth of thermal conductivity (Table S4). However, it was noted [66] that the thermal conductivity coefficient could fall considerably, especially at high temperatures, after some time, due to the instability of the colloidal solution. Therefore, additional studies of a test material must be carried out for the correct comparison as the sol preparation and measurement techniques affect the experimental thermal conductivity [67]. Thus, for our systems, essential previous findings are those where the TMA brand was studied [27], the increase in thermal conductivity with the size was shown for the silicon oxide sols of the same manufacturer [28], and the interlaboratory study included TM-50 sample measurements [29]. In this work, the classic Maxwell’s medium theory agreed with the experimental data.

Table A2 sums up the experimental data for all the brands of silicon oxide sols used in this study (Supplementary Materials, Table S5); measurements were carried out for the original samples and the samples were diluted 2, 5, and 10 times. In each case, a linear dependence of the thermal conductivity vs. volume fraction was observed; see Table A2. The theoretical thermal conductivity of silicon oxide nanoparticles is 1.38 W m^−1^ K^−1^ [68]; the nominal value of $k_{NP}$ that gives a slight (up to 1.5–4%) difference between the theory (Maxwell’s) and the experiment is 1.22 W m^−1^ K^−1^; see Figure A5. Figure A6 shows that the temperature dependence of the sol thermal conductivity is similar to that for water but shifted up.

**Table A2:** The experimental values of thermal conductivity (± 0.005) of silicon oxide sols of various brands at 25 °C on a FOX 50 analyzer; in brackets (%), are increases relative to water (*k* = 0.607 W m^−1^ K^−1^). The equation of linear dependence of thermal conductivity on the volume fraction of nanoparticles.

AM	φ,%	1.6	3.2	8.1	16.1	$k=4.7\cdot 10^{-3}\varphi +0.607$, *R*^2^ = 0.999
	*k*, W m^−1^ K^−1^	0.615	0.621 (2)	0.646 (6)	0.682 (12)	
SM-30	φ,%	1.6	3.2	8.1	16.1	$k=4.1\cdot 10^{-3}\varphi +0.604$, *R*^2^ = 0.998
	*k*, W m^−1^ K^−1^	0.611	0.619 (2)	0.636 (5)	0.672 (10)	
TMA	φ,%	1.9	3.8	9.4	18.8	$k=4.1\cdot 10^{-3}\varphi +0.613$, *R*^2^ = 0.990
	*k*, W m^−1^ K^−1^	0.621	0.626 (3)	0.656 (8)	0.688 (13)	
HS-40	φ,%	2.3	4.6	11.5	23	$k=4.4\cdot 10^{-3}\varphi +0.613$, *R*^2^ = 0.993
	*k*, W m^−1^ K^−1^	0.619	0.638 (5)	0.665 (9)	0.714 (18)	
CL-X	φ,%	2.7	5.4	13.4	26.9	$k=3.5\cdot 10^{-3}\varphi +0.608$, *R*^2^ = 0.998
	*k*, W m^−1^ K^−1^	0.616	0.628 (3)	0.657 (8)	0.703 (16)	
TM-50	φ,%	3.1	6.2	15.4	30.9	$k=4.8\cdot 10^{-3}\varphi +0.607$, *R*^2^ = 0.999
	*k*, W m^−1^ K^−1^	0.622	0.636 (5)	0.681 (12)	0.755 (24)	

An increase in thermal conductivity against water for medium concentrations (3.2–6.2% *v*/*v*) was 2–5%, consistent with the previous studies [27,64,65]. The increase for the TMA brand was 13% for the initial solution, which is lower than the data [27], where the hot-wire method was used. Although with such a very concentrated colloidal solution all the drawbacks of contact methods [11,69,70] may appear, the data for the more diluted solutions can be more reliable. However, it is not easy to compare them as the authors present them only in a figure that shows increases relative to water [27,28]. For the initial solution of the TM-50 brand, the experimental thermal conductivity is 0.755 ± 0.005 W m^−1^ K^−1^, which satisfactorily agrees with the previous data, 0.728 ± 0.007 W m^−1^ K^−1^ [29].
