# Supplementary material for: Thermal Conductivity of Detonation Nanodiamond Hydrogels and Hydrosols by Direct Heat Flux Measurements
Source: Gels. 2021 Dec 3;7(4):248. doi: 10.3390/gels7040248 (PMC8701344; doi:10.3390/gels7040248)
Supplement: Supplementary file 1 [file gels-07-00248-s001.zip › gels-1475691-supplementary.pdf]

Supplementary Materials

Thermal Conductivity of Detonation Nanodiamond Hydrogels and Hydrosols by Direct Heat Flux Measurements

Liliya O. Usoltseva, Dmitry S. Volkov, Mikhail V. Korobov, and Mikhail A. Proskurnin

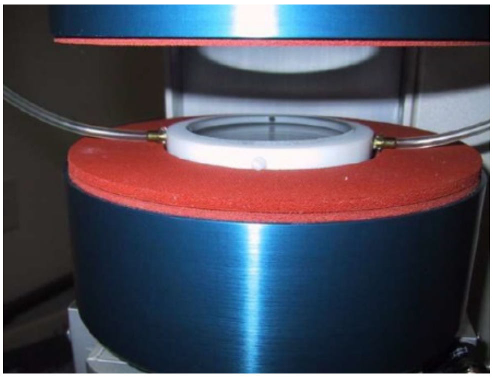

Figure S1. Cell for fluids of the FOX 50 analyzer.

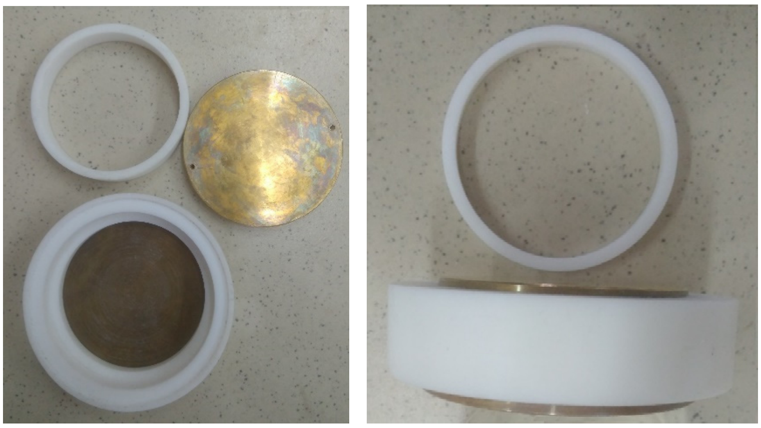

Figure S2. Cell for paste specimens (top view) in a disassembled form (left) and a side view with a thin spacer (right).

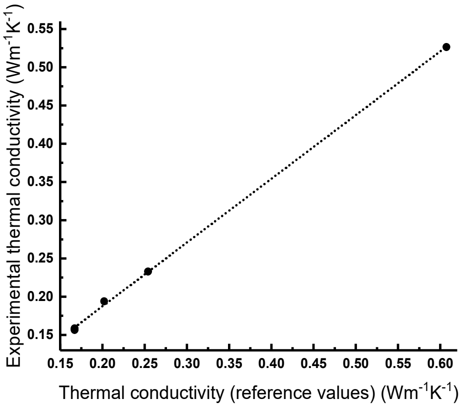

Figure S3. Linear (R^2 = 0.999) dependence of the experimental and reference thermal conductivity values for NMP, ethanol, methanol, ethylene glycol, and water.

S1. Characterization of detonation nanodiamonds

S1.1. Particle size

The paper used the only approach for characterization of particle sizes based on the DSC method since correlations with the results of other methods were obtained in advance. Thus, the X-ray diffraction method was used to determine the sizes of primary crystallites. The survey diffractograms for the RDDM, SDND, RUDDM brands contain three diffraction maxima at angles 2Θ, 43.9; 75.3 and 91.5, corresponding to the crystallographic planes (111), (220) and (311) of the cubic diamond crystal lattice. All the samples studied are diamonds [1,2]. No extraneous phases were detected in significant amounts (more than 5 %). Based on the broadening effect of the X-ray diffraction line, the size of crystallites for SDND and RUDDM were determined as 3.1 ± 0.2 and 3.8 ± 0.2 nm, respectively. The sizes obtained by the X-ray diffraction method for the initial samples and obtained during fractionation correlate well with the data of TEM and DSC, which is most sensitive to size changes [1].

The DLS method was used to determine the size of clusters of colloidal solutions of various brands; it is seen (Fig. S4) the correspondence between the parameter ΔT and the size of aggregates in solution [1], i.e., a correlation between the particle sizes in powders, and the size of aggregates in aqueous dispersions prepared from them was demonstrated. Furthermore, a similar correlation for fractions was observed for the hydration radius obtained from small-angle neutron scattering data [3,4].

Table S1. Nanodiamond brands used in the study and estimated mean particle sizes from the Gibbs–Kelvin equation (P = 0.95, n = 3).

NDs brand	Description	Manufacturer	DSC measurements with the Gibbs–Kelvin equation	
			ΔT, °C	Mean particle diameter, nm
SDND	Single-Digit NanoDiamonds	PlasmaChem GmbH, Berlin, Germany	9.0 ± 0.3	7.3 ± 0.5
RUDDM	modified nanodiamond material of RUDDM grade, fraction 0–150	Real-Dzerzhinsk LLC, Dzerzhinsk, Russia	7.5 ± 0.5	10.1 ± 0.7
RDDM	modified nanodiamond material of RDDM grade, fraction 0–0.125		2.8 ± 0.5	25 ± 1

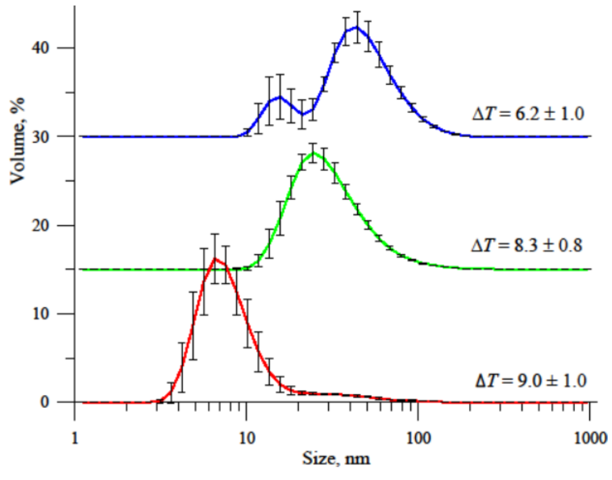

Figure S4. DLS curves for SDND, NanoAmando, and RUDDM samples (red, green, and blue lines). The concentrations of nanodiamonds for all samples were 1.0 mg mL^{-1} [1].

S1.2. Electrokinetic potential (PALS technique)

The potential of aqueous solutions (1 mg mL^{-1}) is –(66.1 ± 1.3), –(64.0 ± 1.1), and –(55 ± 1) mV for RDDM, SDND, and RUDDM, respectively. The pH of aqueous solutions of RDDM, SDND, RUDDM were 5.4, 9.1, and 8.2 (± 0.2). With a decrease in pH, SDND and RUDDM solutions were unstable. With an increase in concentration (2.5 mg mL^{-1}), the potential also decreased –(52.8 ± 1.0) and –(46.3 ± 0.3) for SDND and RUDDM; however, in any case, the studied brands were characterized by high stability, and the sedimentation and aggregative stability of sols weakly correlated with the primary size of crystallites.

S1.3. Impurity composition (ICP-AES)

Table S2. Elements contents in nanodiamonds samples, determined directly in dispersions (all concentrations are in ppm, results uncertainty is ±15%). *1–3: RDDM, RUDDM, SDND. «–» means < 1 μg g^{-1}.

	**1***	**2**	**3**
Ag	-	-	660 ± 100
Al	4 ± 1	300 ± 45	530 ± 80
B	300 ± 45	-	540 ± 80
Ba	-	-	25 ± 4
Bi	-	-	3.0 ± 0.5
Ca	-	78 ± 12	280 ± 40
Cd	-	-	-
Ce	-	-	7 ± 1
Co	-	1.0 ± 0.2	1.9 ± 0.3
Cr	15 ± 2	57 ± 9	11 ± 2
Cu	33 ± 5	410 ± 62	20 ± 3
Fe	133 ± 20	4,100 ± 620	1,780 ± 270
Hf	-	-	-
Hg	-	-	-
K	-	-	20 ± 3
Mg	-	80 ± 1.2	40 ± 6
Mn	-	5 ± 1	21 ± 3
Mo	4 ± 1	12 ± 2	13 ± 2
Na	80 ± 1.2	4,660 ± 700	10,800 ±1,600
Ni	86 ± 13	170 ± 26	15 ± 2
P	-	-	11 ± 2
Pb	52 ± 8	55 ± 8	5 ± 1
S	24 ± 4	80 ± 12	38 ± 6
Sb	-	-	10 ± 2
Si	180 ± 27	170 ± 26	1,470 ± 220
Sn	-	4 ± 1	170 ± 25
Sr	-	-	5 ± 1
Ti	51 ± 8	174 ± 26	285 ± 40
V	-	-	-
W	11 ± 2	4 ± 1	60 ± 9
Y	-	-	-
Zn	4 ± 1	8.0 ± 1.2	3.0 ± 0.5
Zr	23 ± 4	4 ± 1	17 ± 3
∑	933 ± 140	10,300 ± 1,500	16,800 ± 2,500

Detonation nanodiamonds consist of 95–98% carbon; all other elements are present at the level of μg g^{-1} and below. Due to the different production technology, there is a variation in the contents and set of elements. The ICP–AES directly determines the content of impurities, and direct injection of a colloidal solution (10 mg mL^{-1}) can be used [5]. The most significant impurity is iron (hundreds and thousands of μg g^{-1}), then (100–1000 μg g^{-1}) the components of the reaction chamber walls and the initiators of the explosion (Ti, Cr, and Cu) and elements present in high concentrations in natural waters (Na, Si, Ca, Al, and S). The remaining elements are present in small amounts (< 100 μg g^{-1}). NDs from different manufacturers contain very different impurities and, even for a single product type, they change from lot to lot.

S1.4. Surface examination (IR spectroscopy)

Nanodiamonds have a surface with a significant area and a large number of functional groups. Due to the absence of standard samples of nanodiamonds with precisely known surface composition, quantitative analysis by IR spectroscopy is complex. It is possible to judge the ratio of the number of groups only by the intensity ratio of peaks in one sample. Typical IR spectra and the ratio of absorption bands are shown in Fig. S5 and Table S2. The effect of strong oxidizing acids on nanodiamonds under the same conditions does not lead to a surface with the same qualitative and quantitative composition of functional groups. In addition, IR spectroscopy is insensitive to changes in the dispersion of nanodiamonds (differences in the composition of surface groups for aggregates of different sizes are minimal), which is shown during fractionation [1].

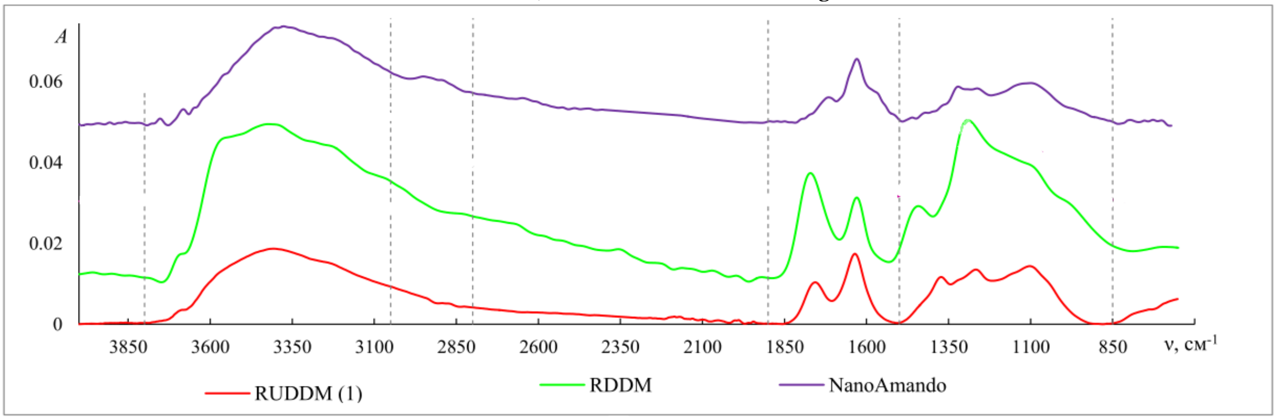

Figure S5. ATR–FTIR spectra in the mid-IR region (4000–650 cm^{-1}) of nanodiamond powders [1,2].

Table S3. Functional groups on the surface of the studied nanodiamonds [2]

Assignment	Band maximum
Hydrogen-bonded –O–H···H_2O stretch	3695
O–H stretch and intermolecular hydrogen bonds	3407
C=O stretch of the anhydride carboxyl group	–
C=O stretch of the isolated carboxyl group	1762
H–O–H bend of liquid adsorbed water	1632
Carboxyl C–O–H in-plane bend	1440 (wide, shoulder)
Non-carboxyl C–O–H in-plane bend	1373
Carboxyl C–O stretch	1267
non-carboxyl C–O stretch	1103
carboxyl out-of-plane C–O–H bend	–

non-carboxyl out-of-plane C–O–H bend	610

S1.5. Pore size (BET)

Powders are formed with a characteristic porous structure consisting of primary nanoparticles combined into agglomerates. The pores are the gaps between the primary particles. The nanodiamond surface has a dual hydrophobic/hydrophilic nature, mesopores are responsible for hydrophobic behavior, and micropores with functional groups are hydrophilic. The surface area of mesopores according to BET for the RUDDM brand is five-fold higher (339 m^2 g^{-1}) than for RDDM (56.7 m^2 g^{-1}), and the micropore area is larger for RDDM (6.9 and 2 m^2 g^{-1}), as well as the average pore diameter: 10.0 and 6.1 nm (for RDDM and RUDDM), which is consistent with the large diameter of primary RDDM particles [2].

S2. Silicon oxide sols

Table S4. Increase in the thermal conductivity of silicon oxide sols relative to water

SiO_2 brand; size, nm*	Volume fraction of nanoparticles, %**	Thermal conductivity increase, %	Temperature,°C	Measurement technique or instrument	Ref.
TM-50, 22	31	21	25	various	[6]
TMA, 32	20	20	22	hot-wire	[7]
SM, HS, TM; 11, 17, 30	16	6, 8, 16	25	hot-wire	[8]
Iolitec, 40 and 80	9.4	5	25	KD2 Pro (hot-wire principle)	[9]
Alfa Aesar, 20	3	28	25	KD2 Pro	[10]
15-20	4	5	25	hot-wire	[11]
Aerosil 200,12	4	2.5	20	3ω	[12]
12	4	23	25	hot-wire	[13]
from bran, 50	3	13.5–38	25–55	KD2 Pro	[14]
30	2.3 (5 w/w)	10–45	20–55	Hot Disk Thermal Constant Analyzer	[15]
12	1	3	25	hot-wire	[16]
XFNANO tech., 20	1	3.4	20	hot wire	[17]
Deke Daojin Sc., 30	1	1	25	KD2 Pro	[18]

* Preparation of a colloidal solution with an ultrasonic bath or probe after adding a powder to water, the first 5 lines — commercial dispersions.

** presents the maximum volume fraction from the studied.

Table S5. Colloidal silicon oxide LUDOX brands AM, SM-30, CL-X, TMA, HS-40, TM-50, (GRACE, USA), the main parameters provided by the manufacturer

Parameter	Brand					
	AM	SM-30	CL-X	TMA	HS-40	TM-50
Surface charge	Negative					
Particle size, nm	12	7	22	22	12	34
Silica (as SiO_2), % w/w	30	30	45	34	40	50
pH (25 °C)	8.9	10.0	9.1	9.0	9.7	9.0
Surface area, m^2 g^{-1}	220	345	130	140	220	140

Titratable Alkali (as Na_2O), % w/w	0.24	0.56	0.19	0.21	0.41	0.5

References

1. Korobov, M.V.; Volkov, D.S.; Avramenko, N.V.; Belyaeva, L.A.; Semenyuk, P.I.; Proskurnin, M.A. Improving the dispersity of detonation nanodiamond: differential scanning calorimetry as a new method of controlling the aggregation state of nanodiamond powders. *Nanoscale* **2013**, *5*, 1529-1536, doi:10.1039/c2nr33512c.
2. Volkov, D.S.; Krivoshein, P.K.; Mikheev, I.V.; Proskurnin, M.A. Pristine detonation nanodiamonds as regenerable adsorbents for metal cations. *Diamond Relat. Mater.* **2020**, *110*, 108121, doi:10.1016/j.diamond.2020.108121.
3. Avdeev, M.; Rozhkova, N.; Aksenov, V.; Garamus, V.; Willumeit, R.; Osawa, E. Aggregate structure in concentrated liquid dispersions of ultrananocrystalline diamond by small-angle neutron scattering. *J. Phys. Chem. C* **2009**, *113*, 9473-9479.
4. Tomchuk, O.V.; Volkov, D.S.; Bulavin, L.A.; Rogachev, A.V.; Proskurnin, M.A.; Korobov, M.V.; Avdeev, M.V. Structural Characteristics of Aqueous Dispersions of Detonation Nanodiamond and Their Aggregate Fractions as Revealed by Small-Angle Neutron Scattering. *J. Phys. Chem. C* **2014**, *119*, 794-802, doi:10.1021/jp510151b.
5. Volkov, D.S.; Proskurnin, M.A.; Korobov, M.V. Elemental analysis of nanodiamonds by inductively-coupled plasma atomic emission spectroscopy. *Carbon* **2014**, *74*, 1-13, doi:10.1016/j.carbon.2014.02.072.
6. Buongiorno, J.; Venerus, D.C.; Prabhat, N.; McKrell, T.; Townsend, J.; Christianson, R.; Tolmachev, Y.V.; Keblinski, P.; Hu, L.-w.; Alvarado, J.L.; et al. A benchmark study on the thermal conductivity of nanofluids. *J. Appl. Phys.* **2009**, *106*, 094312, doi:10.1063/1.3245330.
7. Eapen, J.; Williams, W.C.; Buongiorno, J.; Hu, L.W.; Yip, S.; Rusconi, R.; Piazza, R. Mean-field versus microconvection effects in nanofluid thermal conduction. *Phys. Rev. Lett.* **2007**, *99*, 095901, doi:10.1103/PhysRevLett.99.095901.
8. Chen, G.; Yu, W.; Singh, D.; Cookson, D.; Routbort, J. Application of SAXS to the study of particle-size-dependent thermal conductivity in silica nanofluids. *J. Nanoparticle Res.* **2008**, *10*, 1109-1114, doi:10.1007/s11051-007-9347-y.
9. Haddad, Z.; Abid, C.; Mohamad, A.; Rahli, O.; Bawazer, S. Natural convection of silica–water nanofluids based on experimental measured thermophysical properties: critical analysis. *Heat and Mass Transf.* **2016**, *52*, 1649-1663.
10. Moldoveanu, G.M.; Huminic, G.; Minea, A.A.; Huminic, A. Experimental study on thermal conductivity of stabilized Al2O3 and SiO2 nanofluids and their hybrid. *Int. J. Heat Mass Transf.* **2018**, *127*, 450-457.
11. Kang, H.U.; Kim, S.H.; Oh, J.M. Estimation of Thermal Conductivity of Nanofluid Using Experimental Effective Particle Volume. *Exp. Heat Transf.* **2006**, *19*, 181-191, doi:10.1080/08916150600619281.
12. Tavman, I.; Turgut, A.; Chirtoc, M.; Schuchmann, H.; Tavman, S. Experimental investigation of viscosity and thermal conductivity of suspensions containing nanosized ceramic particles. *Arch. Mater. Sci.* **2008**, *100*.
13. Jahanshahi, M.; Hosseinizadeh, S.; Alipanah, M.; Dehghani, A.; Vakilinejad, G. Numerical simulation of free convection based on experimental measured conductivity in a square cavity using Water/SiO2 nanofluid. *Int. Commun. Heat Mass Transf.* **2010**, *37*, 687-694.
14. Ranjbarzadeh, R.; Moradikazerouni, A.; Bakhtiari, R.; Asadi, A.; Afrand, M. An experimental study on stability and thermal conductivity of water/silica nanofluid: Eco-friendly production of nanoparticles. *J. Clean. Prod.* **2019**, *206*, 1089-1100.
15. Yan, S.; Wang, F.; Shi, Z.; Tian, R. Heat transfer property of SiO2/water nanofluid flow inside solar collector vacuum tubes. *Appl. Therm. Eng.* **2017**, *118*, 385-391.
16. Hwang, Y.; Ahn, Y.; Shin, H.; Lee, C.; Kim, G.; Park, H.; Lee, J. Investigation on characteristics of thermal conductivity enhancement of nanofluids. *Curr. Appl. Phys.* **2006**, *6*, 1068-1071.
17. Guo, W.; Li, G.; Zheng, Y.; Dong, C. Measurement of the thermal conductivity of SiO2 nanofluids with an optimized transient hot wire method. *Thermochim. Acta* **2018**, *661*, 84-97.
18. Ahmed, M.; Yusoff, M.Z.; Ng, K.C.; Shuaib, N. Numerical and experimental investigations on the heat transfer enhancement in corrugated channels using SiO2–water nanofluid. *Case Stud. Therm. Eng.* **2015**, *6*, 77-92.
